# Work‐Related Information and Communication Technologies Use After‐Hours and Nurse Work Withdrawal Behavior: A Moderated Mediation Model

**DOI:** 10.1155/jonm/8204893

**Published:** 2026-09-07

**Authors:** Xiaofen Wang, Yichen Geng, Jingwen Yi, Danyan Li, Miliang Zou, Haiyan Liu, Kun Tang

**Affiliations:** ^1^ School of Nursing, Health Science Center, Hunan Normal University, Changsha, Hunan, China, hunnu.edu.cn; ^2^ Department of Oncology, The Second Xiangya Hospital of Central South University, Changsha, Hunan, China, csu.edu.cn; ^3^ Hemodialysis Center, The Third Hospital of Changsha, Changsha, Hunan, China; ^4^ Department of Comprehensive Evaluation Center (Quality Management), Xiangya Hospital of Central South University, Changsha, Hunan, China, csu.edu.cn

**Keywords:** job burnout, nurses, psychological detachment, W_ICTs, work withdrawal behavior

## Abstract

**Background:**

The widespread adoption of information technology made the work‐related use of information and communication technologies after hours (W_ICTs) a prevalent source of occupational stress among nurses. Grounded in conservation of resources theory and the effort‐recovery model, this study constructed a moderated mediation model to examine the impact of W_ICTs on nurses’ work withdrawal behavior, with a specific focus on the mediating role of job burnout and the moderating role of psychological detachment.

**Methods:**

Using a cross‐sectional design, data were collected through an online survey between September and December 2025. A total of 869 clinical nurses (mean age = 32.47 ± 5.95 years) from 10 tertiary hospitals in China completed validated scales measuring W_ICTs, job burnout, psychological detachment, and work withdrawal behavior. Data were analyzed using SPSS 26.0 and the PROCESS macro (Model 8), controlling for gender, age, and professional title. A moderated mediation model was tested.

**Results:**

W_ICTs showed a direct positive association with work withdrawal behavior and an indirect association through job burnout. This mediating effect was consistent for both challenge‐oriented and hindrance‐oriented communications. Psychological detachment significantly moderated the relationship between W_ICTs and job burnout, attenuating the effect of challenge‐oriented W_ICTs but not hindrance‐oriented W_ICTs.

**Conclusion:**

Job burnout mediates the effect of W_ICTs on nurses’ work withdrawal behavior. Psychological detachment moderates this relationship for challenge‐oriented but not hindrance‐oriented communication. Nursing managers should therefore implement strategies to reduce hindrance‐oriented W_ICTs, schedule challenge‐oriented communications judiciously, and strengthen nurses’ psychological detachment through systematic interventions to reduce job burnout and work withdrawal behavior.

**Implications for the Nursing Management:**

Reducing W_ICTs, particularly those that are hindrance‐oriented, and enhancing nurses’ psychological detachment, can reduce job burnout and work withdrawal behavior. Findings inform targeted management strategies to protect nurse well‐being and retention in the digital age.

## 1. Introduction

The pervasive integration of electronic communication tools—such as mobile phones and computers—into workplace communication has become a defining feature of modern healthcare. While these digital tools reduce organizational communication costs and transcend temporal–spatial boundaries, they simultaneously blur the traditional lines between professional and personal lives [1]. This phenomenon is particularly acute within the nursing profession, where clinical responsibilities frequently extend beyond scheduled shifts [2]. Nurses often receive work‐related messages from colleagues, supervisors, and patients during evenings, weekends, holidays, and designated rest periods via telephone, instant messaging, or email. This practice is termed work‐related use of information and communication technologies after hours (W_ICTs) [3, 4]. Despite its role in operational efficiency, its intrusive nature has evolved into a widespread occupational stressor [4, 5].

Although early European Union directives established “work‐free periods” to safeguard employee recovery time [6], W_ICTs remain prevalent among nurses globally, including in China. Against the backdrop of international nursing shortages and escalating healthcare demands, nurses frequently manage overwhelming workloads by sacrificing personal rest, often without compensatory benefits [7, 8]. Evidence indicates that exposure to W_ICTs elevates risks to physical health and increases psychological strain [9]. Persistent after‐hours work encroaches on sleep and leisure, disrupts self‐repair mechanisms, and hinders complete psychological detachment from professional roles, leading to a state of sustained cognitive activation [5]. This chronic depletion of psychological resources is associated with job burnout and has been linked to job satisfaction and organizational commitment [9, 10], establishing W_ICTs as a critical concern in nursing occupational health research.

Emerging research, however, suggests that the impact of W_ICTs is not uniform; rather, it varies significantly depending on the communication’s nature [11]. Applying the challenge–hindrance stress framework [12], W_ICTs can be appraised as either challenge‐oriented (e.g., communications involving problem‐solving, learning, or professional development) or hindrance‐oriented (e.g., those involving criticism, unnecessary complications, or ambiguous demands). These distinct stressor types are theorized to elicit divergent psychological and behavioral responses, with hindrance stressors typically demonstrating stronger negative associations with well‐being and job attitudes. However, the empirical understanding of these differential effects, particularly within nursing, remains limited.

Employees who experience job dissatisfaction or adverse work conditions may engage in work withdrawal behavior as a coping strategy to reduce negative affect [13]. This construct encompasses both psychological and behavioral dimensions. Psychological withdrawal involves internal disengagement, such as diminished commitment, apathy toward duties, reduced empathy, and mental detachment during work [4]. Behavioral withdrawal includes observable actions like tardiness, absenteeism, minimized interactions, or new job searching [13]. Research confirms that W_ICTs are associated with fatigue and work withdrawal [4, 14]. Notably, in China, 37.3% of nurses report psychological withdrawal, and 13.7% exhibit moderate‐to‐severe behavioral withdrawal [15]. These behaviors undermine care quality, increase medical errors, weaken teamwork, and raise turnover, ultimately threatening patient safety and organizational stability [16, 17].

Job burnout, a state of physical and emotional exhaustion from chronic work stress, is highly prevalent in nursing [18]. In high‐intensity environments, sustained job burnout jeopardizes well‐being and career continuity, sometimes leading to resignation [19]. W_ICTs have been suggested as a potential antecedent of nursing job burnout, as the “always‐on” pattern impedes psychological recovery, depletes emotional reserves, and fosters work alienation [5]. According to Conservation of Resources Theory, individuals adopt defensive strategies to conserve resources when faced with persistent loss [20]. Thus, nurses experiencing job burnout may employ work withdrawal as a self‐protective mechanism to conserve remaining resources. This positions job burnout as a plausible critical mediator in the pathway linking W_ICTs to work withdrawal behavior. Nevertheless, the specific mediating pathways and boundary conditions remain inadequately understood, particularly concerning protective factors and the distinct effects of challenge‐versus hindrance‐oriented W_ICTs.

Psychological detachment refers to a positive restorative experience involving mental disengagement from work during nonwork hours [21]. It allows individuals to consciously “switch off,” avoid work‐related rumination, and redirect attention to personal life [22]. This capacity is associated with lower emotional exhaustion, greater engagement, improved performance, and enhanced job satisfaction across professions [23, 24]. For nurses, in particular, psychological detachment has been shown to reduce job burnout and post‐traumatic stress symptoms and to buffer the stress and fatigue associated with intrusive after‐hours communications [25, 26]. Furthermore, higher levels of detachment correlate with fewer withdrawal behavior and are linked to more positive perceptions of patient safety [27]. These findings collectively suggest that psychological detachment may reduce the adverse effects of W_ICTs on job burnout and work withdrawal behavior among nurses. However, whether this moderating role varies between challenge‐oriented and hindrance‐oriented W_ICTs remains unexamined.

Conservation of Resources Theory posits that individuals strive to acquire and protect their valuable resources (e.g., time, energy, and emotions) [20]. When these resources are threatened, stress such as job burnout arises, prompting defensive actions like work withdrawal to prevent further loss. Complementary to this, the Effort‐Recovery Model emphasizes that effective recovery from work stressors (e.g., through psychological detachment) is essential for sustaining long‐term well‐being [28]. Within this theoretical framework, W_ICTs represent a persistent drain on personal resources—a prototypical resource depletion event that may be associated with nurses′ job burnout and subsequent withdrawal behavior. Job burnout, as the core manifestation of resource depletion, is thus hypothesized to mediate the relationship between W_ICTs and work withdrawal. Concurrently, psychological detachment, as a vital recovery experience, is proposed to moderate the pathway from W_ICTs to job burnout, with its attenuating effect potentially varying based on whether the W_ICTs are challenge‐oriented or hindrance‐oriented.

**FIGURE 1:**
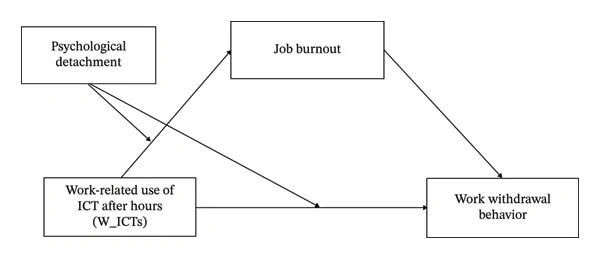
The conceptual framework of the hypothesis. W_ICTs: work‐related use of information and communication technologies after hours.

Integrating these theoretical perspectives, this study proposes a moderated mediation model (see Figure 1). The research hypotheses are as follows:

 H1: W_ICTs are positively correlated with nurses′ work withdrawal behavior, with job burnout mediating this relationship.
 H2a: Psychological detachment attenuates the positive effect of W_ICTs on job burnout.
 H2b: Psychological detachment attenuates the positive effect of W_ICTs on work withdrawal behavior.
 H3 (exploratory): The moderating effect of psychological detachment may differ between challenge‐oriented and hindrance‐oriented W_ICTs.

The findings may offer novel insights into the mechanisms of work‐related stress transmission among nurses, provide empirical support for safeguarding nursing occupational health, and inform the design of targeted future interventions.

## 2. Materials and Methods

### 2.1. Study Design

This study was conducted as a multicenter, cross‐sectional survey between September and December 2025. A convenience sample of nurses was recruited from 10 tertiary general hospitals across Hunan, Guangxi, Jiangxi, and Hubei provinces in China. Data were collected using an electronic questionnaire administered via the Wenjuanxing platform. With the assistance of hospital nursing departments or ward supervisors, the questionnaire link was distributed during nursing meetings, training breaks, or through internal work group chats. Prior to participation, all potential respondents were provided with a uniform explanation of the study’s objectives, the principles of anonymity, and data confidentiality. This study was reported in accordance with the Strengthening the Reporting of Observational Studies in Epidemiology (STROBE) Statement for cross‐sectional studies.

### 2.2. Setting and Participants

Participation was voluntary and contingent upon informed consent. Inclusion criteria were (1) being a registered nurse with a valid practicing certificate; (2) being directly engaged in clinical nursing with at least 1 year of working experience; (3) routinely using smartphones or computers for work‐related electronic communication; and (4) providing informed consent for voluntary participation. Exclusion criteria included (1) nurses on extended leave (e.g., maternity or sick leave); (2) nurses in nonclinical frontline roles (e.g., management, education); and (3) interns, trainees, or resident nurses. The sample size was calculated using the formula: *n* = *Z* ^2^ *P*(1 − *P*)/*δ* ^2^, where *Z* = 1.96, *δ* = 0.05, and *P* (the estimated prevalence of nursing withdrawal behavior) was set at 51% based on prior research [15]. This yielded a minimum sample size of 384. Accounting for a design effect of 1.5 and an estimated 15% nonresponse rate, the final planned sample size was 678. All questionnaire items were mandatory. To ensure data quality, responses were screened and excluded if they met any of the following criteria: (1) completion in less than 50% of the standard estimated time; (2) patterned responses (e.g., selecting the same option for all questions); or (3) containing obvious logical inconsistencies.

### 2.3. Measures

#### 2.3.1. General Information Questionnaire

A self‐designed questionnaire was employed to collect demographic and professional characteristics of nurses, including age, gender, educational level, marital status, years of service, professional title, and clinical department.

#### 2.3.2. W_ICTs

The Chinese version of the W_ICTs scale [29] was employed to evaluate the use of W_ICTs by nurses. This scale consists of three items rated on a 5‐point Likert scale (1 = never to 5 = very frequently), with a total score range of 3–15; higher scores indicate more frequent W_ICTs use. The scale has shown good reliability in Chinese samples (Cronbach’s *α* = 0.740∼0.800); in the present study, *α* was 0.824.

Given the lack of an established instrument specifically designed to classify after‐hours work communication into challenge‐oriented versus hindrance‐oriented types, the present study adopted a theory‐driven approach based on the challenge–hindrance stress framework [12] to develop two supplementary items to capture the core characteristics of each communication type. The specific items were (1) “After hours, I receive work‐related communications that involve problem‐solving, learning, or professional development” (challenge‐oriented); and (2) “After hours, I receive work‐related communications that involve criticism, unnecessary complications, or ambiguous demands” (hindrance‐oriented). Both items are rated on the same 5‐point Likert scale ranging from 1 (never) to 5 (very frequently). These two items were designed as classification indicators to categorize participants based on a difference score (challenge minus hindrance), rather than as a multi‐item measurement scale. The appropriateness of the item content was reviewed by five experts in occupational health and nursing research. The experts rated whether each item adequately reflected the core characteristics of its respective communication type as distinguished by the challenge–hindrance stress framework. The agreement among the experts was high (0.906), indicating that the items were judged by the experts to be appropriate representations of the two communication types (Cronbach’s *α* was not computed for these two items, as they were not intended to measure a single latent construct).

A difference score (challenge − hindrance) was then calculated; participants with a score > 0 were classified into the challenge‐oriented group, and those with a score < 0 into the hindrance‐oriented group. Separate moderated mediation analyses were then conducted for each group. This classification approach allowed us to compare the moderated mediation pathways between the two subgroups while keeping the dependent variable (the W_ICTs total score) consistent across all analyses, thereby ensuring comparability with the overall sample findings.

#### 2.3.3. Job Withdrawal Behavior

The Job Withdrawal Behavior Scale (JWBS) developed by Lehman et al. [13] was employed to assess nurses′ work withdrawal behavior. This 12‐item scale includes 8 items measuring psychological withdrawal and 4 items measuring behavioral withdrawal, each rated on a 5‐point Likert scale from 1 (strongly disagree) to 5 (strongly agree). Total scores range from 12 to 60, with higher scores indicating more severe withdrawal behavior. Cronbach’s *α* coefficient for the Chinese version of this scale is 0.900, and in this study, it was 0.926.

#### 2.3.4. Job Burnout

The Chinese version of the Copenhagen Burnout Inventory (CBI), revised by Wu et al. [30], was employed to assess nurses′ job burnout. The scale includes 19 items across three dimensions: personal burnout, work‐related burnout, and patient or colleague burnout. Responses are recorded on a 5‐point Likert scale (1 = never to 5 = always), with total scores ranging from 19 to 95; higher scores reflect higher levels of job burnout. The scale has demonstrated strong reliability in previous studies (total Cronbach’s *α* = 0.936; subscale Cronbach’s *α* = 0.853∼0.932). In the current sample, Cronbach’s *α* was 0.934.

#### 2.3.5. Psychological Detachment

Psychological detachment was measured using the Chinese version of the Psychological Detachment Scale (PDS) developed by Sonnentag et al. [21] and adapted by Lu et al. [31]. This 4‐item unidimensional scale (e.g., “After work, I forget about work‐related matters”) uses a 5‐point Likert scale from 1 (strongly disagree) to 5 (strongly agree). Total scores range from 4 to 20, with higher scores indicating greater ability to mentally disengage from work. The scale has been widely used in nursing research with good reliability (original Cronbach’s *α* = 0.833; current study Cronbach’s *α* = 0.896).

### 2.4. Data Analysis

Data were analyzed using SPSS 26.0 and the PROCESS macro (version 4.1). Descriptive statistics, correlation analysis, and common method bias assessment were first performed. To select control variables, a multiple linear regression was conducted with work withdrawal behavior as the dependent variable and general information variables as predictors. Working years were excluded due to multicollinearity with age. For the retained variables, variance inflation factors (VIFs) ranged from 1.12 to 1.94, indicating no concerns for multicollinearity. Variables significantly associated with work withdrawal behavior (*p* < 0.05)—namely, age, gender, and professional title—were retained as covariates in subsequent analyses. The subgroup analyses comparing challenge‐oriented and hindrance‐oriented W_ICTs groups were exploratory in nature and were not prespecified as formal hypotheses. Hayes’ PROCESS Model 4 was used to examine the mediating role of job burnout in the relationship between W_ICTs and work withdrawal behavior. PROCESS Model 8 was applied to test whether psychological detachment moderated the direct and/or indirect effects of W_ICTs, with significant interaction effects probed via simple slope analysis. All continuous predictors were mean‐centered prior to analysis, and results are reported using unstandardized regression coefficients. Standardized indirect effects are additionally reported for the hindrance‐oriented subgroup analysis, where the interaction term was not significant, allowing for a simple mediation interpretation. Bootstrap confidence intervals (95% CI) were calculated using 5000 bootstrap samples, with *p* < 0.05 indicating statistical significance.

### 2.5. Ethical Considerations

The research protocol was reviewed and approved by the Ethics Committee of Hunan Normal University (Approval No.: 2025‐899). A detailed description of the study purpose, procedures, and participants’ rights was provided on the cover page of the questionnaire. For the online survey, informed consent was obtained electronically when participants clicked “I agree to participate in this survey.”

## 3. Results

### 3.1. Common Method Variance Assessment

Common method variance was assessed using Harman’s single‐factor test, which was conducted through unrotated principal component analysis including all study variables (W_ICTs, job burnout, work withdrawal behavior, and psychological detachment). The analysis extracted 10 components (eigenvalues > 1), wherein the first component accounted for 22.36% of the total variance—well below the 40% threshold [32]. These findings indicate that no significant common method variance exists in the current study.

To further assess the potential influence of common method variance, we conducted a common latent factor (CLF) analysis following the recommended procedures of Podsakoff et al. [32]. The CLF accounted for 20.48% of the total variance across all measured indicators, which is substantially below the recommended threshold of 50%. After controlling for the CLF, the path coefficients between substantive constructs changed only marginally, with the largest difference being 0.13—well below the 0.20 benchmark suggested as indicative of meaningful method effects. These findings further confirm that common method variance does not pose a serious threat to the validity of our findings.

### 3.2. Descriptive Statistics and Correlation Analysis

Of the 920 nurses who initially responded, 51 were excluded due to excessively rapid completion (*n* = 19), highly consistent response patterns (*n* = 20), or logical inconsistencies (*n* = 12). The final analytic sample comprised 869 participants. The sample was predominantly female (87.7%), with a mean age of 32.47 (±5.95) years. The most common educational background was a bachelor’s degree (59.7%), and the most frequent professional title was staff nurse (38.2%). Based on the W_ICTs difference score, 343 participants (39.5%) were classified as the challenge‐oriented W_ICTs group and 526 (60.5%) as the hindrance‐oriented W_ICTs group. For exploratory purposes, the 12 participants with a difference score of 0 were randomly allocated to the two groups (6 to each); sensitivity analyses excluding these participants were subsequently conducted to verify the robustness of the subgroup findings. Detailed demographic and professional characteristics of the sample are presented in Table 1.

**TABLE 1:** Sample characteristics (*n* = 869).

Variables		Frequency	Percentage (%)
Age (years)	21∼48 (32.47 ± 5.95)	—	—

Working experience (years)	1∼28 (9.08 ± 3.43)	—	—

Gender	Male	107	12.3
	Female	762	87.7

Marital status	Unmarried	294	33.8
	Married	507	58.3
	Divorced/Widowed	68	7.8

Educational level	College or below	169	19.4
	Bachelor’s degree	519	59.7
	Master’s degree or above	181	20.8

Professional title	Nurse	219	25.2
	Staff nurse	332	38.2
	Nurse‐in‐charge	274	31.5
	Associate chief nurse or above	44	5.1

Department	Internal medicine	277	31.9
	Surgery	221	25.4
	Specialized units	288	33.1
	Medical technology/Outpatient	83	9.6

W_ICTs group	Challenge‐oriented	343	39.5
	Hindrance‐oriented	526	60.5

*Note:* W_ICTs: Work‐related use of information and communication technologies after hours.

As presented in Table 2, the Pearson correlation analysis indicated that W_ICTs were positively correlated with both job burnout (*r* = 0.688, *p* < 0.01) and work withdrawal behavior (*r* = 0.737, *p* < 0.01). Job burnout was also positively correlated with work withdrawal behavior (*r* = 0.839, *p* < 0.01). Conversely, psychological detachment showed significant negative correlations with W_ICTs (*r* = −0.687, *p* < 0.01), job burnout (*r* = −0.764, *p* < 0.01), and work withdrawal behavior (*r* = −0.822, *p* < 0.01).

**TABLE 2:** Descriptive statistics and intercorrelations between variables (*n* = 869).

Variables	Mean	SD	1	2	3	4
1. W_ICTs	12.12	2.62	1	—	—	—
2. Job burnout	40.97	10.24	0.688^∗∗^	1	—	—
3. Psychological detachment	10.03	4.50	−0.687^∗∗^	−0.764^∗∗^	1	—
4. Work withdrawal behavior	29.90	10.37	0.737^∗∗^	0.839^∗∗^	−0.822^∗∗^	1

*Note:* W_ICTs: Work‐related use of information and communication technologies after hours.

^∗∗^ *p* < 0.01.

### 3.3. Moderated Mediation Analysis

Prior to the main analyses, regression diagnostics were performed to assess model assumptions. Residual analysis and the Durbin–Watson test supported the assumptions of linearity, homoscedasticity, and independence of errors (Durbin–Watson = 1.91 for the job burnout model and 2.03 for the work withdrawal model). Effect sizes are reflected by the *R* ^2^ values reported in Table 3.

**TABLE 3:** Results of the moderated mediation analysis (*n* = 869).

Outcome variable	Predictor variable	*R*	*R* ^2^	*B*	SE	95% CI	*t*	*p*
Job burnout		0.812	0.659					
	Age			−0.006	0.004	(−0.014 0.002)	−1.383	0.167
	Gender			0.221	0.066	(0.092 0.351)	3.349	0.001
	Professional title			0.107	0.045	(0.019 0.196)	2.382	0.017
	W_ICTs			0.400	0.030	(0.340 0.459)	13.103	< 0.001
	Psychological detachment			−0.483	0.029	(−0.540 −0.426)	−16.689	< 0.001
	W_ICTs × Psychological detachment			−0.174	0.025	(−0.223 −0.126)	−7.104	< 0.001

Work withdrawal behavior		0.895	0.801					
	Age			−0.001	0.003	(−0.007 0.006)	−0.172	0.864
	Gender			0.004	0.051	(−0.096 0.105)	0.086	0.931
	Professional title			0.040	0.035	(−0.028 0.108)	1.157	0.248
	W_ICTs			0.183	0.026	(0.132 0.234)	7.109	< 0.001
	Job burnout			0.433	0.029	(0.377 0.489)	15.173	< 0.001
	Psychological detachment			−0.360	0.028	(−0.415 −0.305)	−12.915	< 0.001
	W_ICTs × Psychological detachment			0.018	0.024	(−0.029 0.065)	0.753	0.452

*Note:* W_ICTs: Work‐related use of information and communication technologies after hours.

The simple mediation analysis, controlling for age, gender, and professional title, indicated that W_ICTs had a significant positive direct effect on job burnout (*B* = 0.65, *p* < 0.001). Both W_ICTs (*B* = 0.30, *p* < 0.001) and job burnout (*B* = 0.62, *p* < 0.001) were positively associated with work withdrawal behavior. The indirect effect of W_ICTs on work withdrawal behavior via job burnout was significant (*B* = 0.40, bootstrap 95% CI [0.37, 0.43]), accounting for 56.8% of the total effect. After introducing psychological detachment into the model, the mediating effect of job burnout remained significant (*B* = 0.17, bootstrap 95% CI [0.14, 0.21]), thus supporting Hypothesis 1. The full moderated mediation results are presented in Table 3.

**FIGURE 2:**
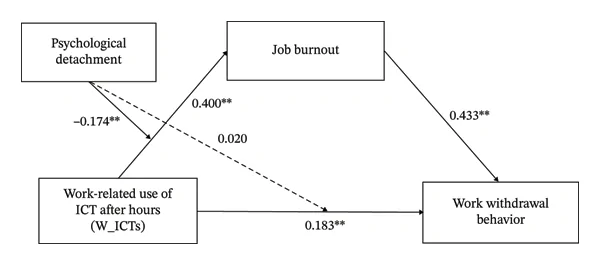
The moderated mediation model based on the total score of W_ICTs (^∗∗^ *p* < 0.001). W_ICTs: work‐related use of information and communication technologies after hours.

Further moderated mediation analysis was conducted using PROCESS Model 8, controlling for age, gender, and professional title. The results showed that psychological detachment was significantly and negatively associated with both job burnout (*B* = −0.48, *p* < 0.001) and work withdrawal behavior (*B* = −0.36, *p* < 0.001). Critically, the interaction term between psychological detachment and W_ICTs showed a significant negative association with job burnout (*B* = −0.17, *p* < 0.001), supporting Hypothesis 2a. However, the interaction term between psychological detachment and W_ICTs was not a significant predictor of work withdrawal behavior (*B* = 0.02, *p* = 0.452); thus, Hypothesis 2b was not supported. The final moderated mediation model is illustrated in Figure 2.

**FIGURE 3:**
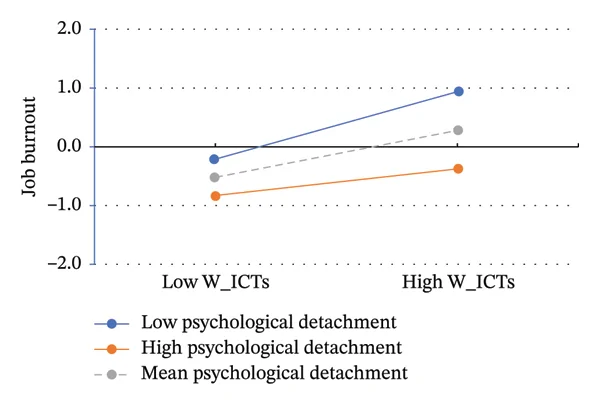
Moderating effect of psychological detachment on the relationship between W_ICTs and job burnout. W_ICTs: work‐related use of information and communication technologies after hours.

Simple slope analysis further clarified this moderating effect (see Figure 3). At low levels of psychological detachment, a one‐unit increase in W_ICTs was associated with a 0.57‐unit increase in job burnout (*B* = 0.57, *p* < 0.001). In contrast, at high levels of psychological detachment, the same increase in W_ICTs corresponded to only a 0.23‐unit increase in job burnout (*B* = 0.23, *p* < 0.001). These results indicate that psychological detachment significantly attenuates the positive relationship between W_ICTs and job burnout.

### 3.4. Moderated Mediation Analysis of Different W_ICTs Group

**FIGURE 4:**
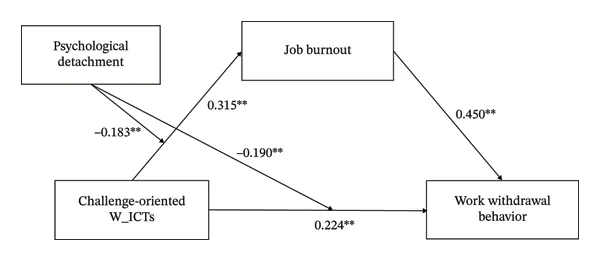
The moderated mediation model based on the challenge‐oriented W_ICTs (^∗∗^ *p* < 0.001). W_ICTs: work‐related use of information and communication technologies after hours.

Separate moderated mediation analyses were conducted for the challenge‐oriented and hindrance‐oriented W_ICTs groups. In the challenge‐oriented group, after controlling for age, gender, and professional title and including psychological detachment in the model, the mediating effect of job burnout between challenge‐oriented W_ICTs and work withdrawal behavior remained significant (*B* = 0.14, bootstrap 95% CI [0.10, 0.18]). Moreover, the interaction between psychological detachment and challenge‐oriented W_ICTs showed significant negative associations with both job burnout (*B* = −0.18, *p* < 0.001) and work withdrawal behavior (*B* = −0.19, *p* < 0.001) (see Figure 4).

**FIGURE 5:**
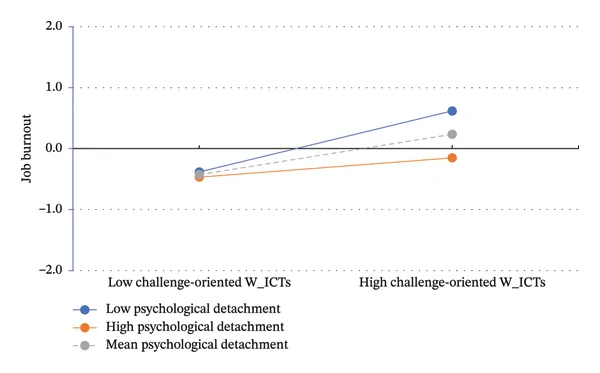
Moderating effect of psychological detachment on the relationship between challenge‐oriented W_ICTs and job burnout. W_ICTs: work‐related use of information and communication technologies after hours.

Simple slope analysis further elucidated these interactions. For job burnout, a one‐unit increase in challenge‐oriented W_ICTs was associated with a 0.48‐unit increase when psychological detachment was low (*B* = 0.48, *p* < 0.001), while the same increase corresponded to only a 0.15‐unit increase when psychological detachment was high (*B* = 0.15, *p* < 0.001). This pattern indicates that psychological detachment significantly attenuated the positive effect of challenge‐oriented W_ICTs on job burnout (see Figure 5).

**FIGURE 6:**
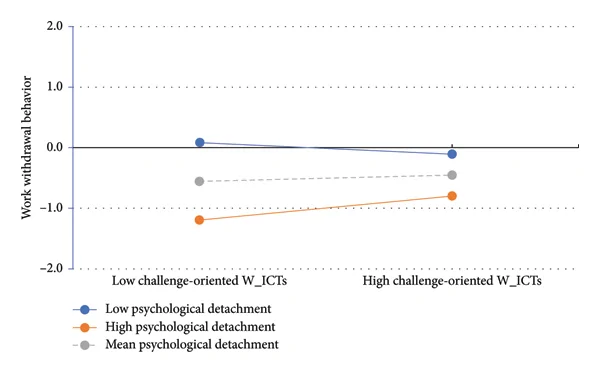
Moderating effect of psychological detachment on the relationship between challenge‐oriented W_ICTs and work withdrawal behavior. W_ICTs: work‐related use of information and communication technologies after hours.

For work withdrawal behavior, the direct effect of challenge‐oriented W_ICTs was nonsignificant at low levels of psychological detachment (*B* = −0.09, *p* = 0.138). However, at high levels of psychological detachment, a one‐unit increase in challenge‐oriented W_ICTs was associated with a 0.19‐unit increase in work withdrawal behavior (*B* = 0.19, *p* < 0.001) (see Figure 6).

**FIGURE 7:**
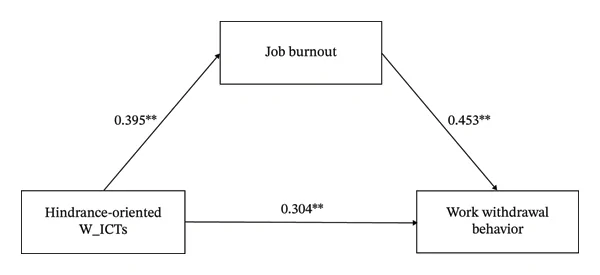
The moderated mediation model based on the hindrance‐oriented W_ICTs (^∗∗^ *p* < 0.001). W_ICTs: work‐related use of information and communication technologies after hours.

The moderated mediation analysis for the hindrance‐oriented W_ICTs group, after controlling for age, gender, and professional title, showed a significant mediating effect of job burnout (*B* = 0.18, bootstrap95%CI [0.13, 0.22]; *β = *0.17, bootstrap 95% CI [0.12, 0.20]). However, the interaction between psychological detachment and hindrance‐oriented W_ICTs was not significant for either job burnout or work withdrawal behavior (both *p* > 0.05) (see Figure 7). These results support Hypothesis 3, indicating that the moderating effect of psychological detachment differs between challenge‐oriented and hindrance‐oriented W_ICTs.

Sensitivity analyses showed that all significant pathways remained stable in both direction and magnitude in both subgroups. Specifically, in the challenge‐oriented group, the mediating effect of job burnout remained significant (*B* = 0.14, bootstrap 95% CI [0.09, 0.18]), and the interaction effects remained significant for both job burnout (*B* = −0.18, *p* < 0.001) and work withdrawal behavior (*B* = −0.19, *p* < 0.001). In the hindrance‐oriented group, the mediating effect of job burnout remained significant (*B* = 0.18, bootstrap 95% CI [0.12, 0.22]; *β = *0.17, bootstrap 95% CI [0.11, 0.20]), and the interaction effects remained nonsignificant for both job burnout and work withdrawal behavior (both *p* > 0.05). These results confirm the robustness of our findings.

## 4. Discussion

Grounded in Conservation of Resources Theory and the Effort‐Recovery Model, this study provides a nuanced examination of how W_ICTs are associated with nurses’ work withdrawal behavior. It represents one of the first investigations in this field to explicitly differentiate between challenge‐oriented and hindrance‐oriented W_ICTs and to test a moderated mediation model incorporating both job burnout and psychological detachment. This integrated theoretical perspective advances understanding of the psychological processes and behavioral responses of nurses in an increasingly digital work environment.

The core finding is that job burnout mediates the relationship between W_ICTs and work withdrawal behavior. Previous research has established links between W_ICTs, employee fatigue, and behavioral withdrawal. Kubo et al. demonstrated that W_ICTs increase work intensity and trigger work‐related rumination, leading to sustained cortisol elevation and fatigue [5]. Wang et al. [33] and Becker et al. [34] found that W_ICTs directly correlate with work–family conflict, triggering individual anxiety and relational tension. Huo et al. confirmed that W_ICTs primarily increase emotional exhaustion by amplifying workplace alienation, thereby inducing employee workplace cheating [35]. Persistent W_ICTs impede resource replenishment, trigger exhaustion, and subsequently foster negative work attitudes, such as reduced engagement [36] and time theft—the unauthorized engagement in nonwork activities during working hours [37]. Employees’ negative responses to W_ICTs also adversely affect their perceptions of leadership efficacy and work attitudes [38]. These findings align with Resource Conservation Theory [20]: W_ICTs, as a persistent resource demand, impede nurses’ psychological recovery and precipitate job burnout. To prevent further depletion, nurses may conserve energy by reducing work engagement—indicating that withdrawal may act as a self‐protective strategy under prolonged resource imbalance.

Psychological detachment further significantly moderates the relationship between W_ICTs and job burnout. This supports Cho et al. ’s research, confirming the protective role of detachment as a key psychological resource in reducing work stress and physical and mental fatigue [22]. According to the Effort‐Recovery Model [28], achieving psychological disconnection during nonwork periods is crucial for interrupting persistent cognitive activation and promoting resource restoration. Sonnentag et al. noted that detachment reduces work‐related rumination and lowers psychophysiological arousal, thereby reducing the adverse effects of prolonged work demands [21]. Allen et al. further demonstrated that high detachment capacity protects nurses’ health and reduces burnout risk, even in stressful situations such as workplace bullying [25]. This highlights the necessity of implementing interventions to enhance nurses′ capacity for psychological detachment.

However, the nature of nursing work presents significant challenges to achieving complete psychological disengagement. Nurses frequently ruminate on specific patient conditions after their shift ends or worry about potential oversights during their duty period. This persistent cognitive engagement leads to continuous depletion of psychological resources within unresolved work contexts [39]. In such contexts, W_ICTs may not only disrupt recovery but also activate work‐related anxiety, creating additional emotional burden and exacerbating job burnout—a vicious cycle of resource loss. Despite these challenges, enhancing nurses’ detachment capacity remains practically important [25]. Healthcare institutions should minimize nonessential W_ICTs while recognizing the regulatory role of psychological detachment. Systematic interventions should therefore aim to strengthen nurses′ recovery capacity and block pathways to work withdrawal behavior.

This study did not find a significant moderating effect of psychological detachment between W_ICTs and work withdrawal behavior. However, detachment exhibited a significant direct negative effect on work withdrawal behavior, suggesting its potential importance in reducing such behavior. This suggests that the protective effect of detachment may operate primarily through alleviating exhaustion rather than directly blocking stress‐induced behavioral responses. When W_ICTs frequently violate personal boundaries, such intrusions may trigger perceptions of organizational unfairness, even among individuals with high detachment capacity [40, 41]. Nurses may perceive W_ICTs as unreasonable demands, achieving psychological “compensatory balance” through withdrawal. This cognitive‐evaluative pathway may operate more directly and independently of detachment compared to the emotional exhaustion pathway. Thus, psychological detachment not only provides indirect protection by reducing job burnout but may also directly inhibit work withdrawal behavior as an independent psychological resource. From the perspective of COR Theory, this dual role reflects both its resource‐protecting function as a buffer against resource depletion and its resource‐preserving function as a personal resource that directly promotes well‐being.

After distinguishing between challenge‐ and hindrance‐oriented W_ICTs, the analysis revealed a more complex pattern. Psychological detachment significantly moderated the relationship between challenge‐oriented W_ICTs and both job burnout and work withdrawal. Specifically, higher detachment weakened the positive effect of challenge‐oriented W_ICTs on job burnout, reflecting its resource value. However, the positive effect of challenge‐oriented W_ICTs on work withdrawal behavior became significant only under high detachment conditions—a seemingly counterintuitive finding that can be understood through boundary theory. Michaelides et al. noted that creating and maintaining work–nonwork boundaries may both reduce conflict by facilitating psychological detachment and increase conflict by depleting self‐control resources [42]. Individuals with high psychological detachment are most successful at boundary management, yet they may also be most sensitive to boundary violations [43]. When challenge‐oriented communications intrude upon rest time—even when the content is inherently “positive”—they may perceive this intrusion itself as a boundary violation, triggering withdrawal as a defensive response to re‐establish control. Yu et al. further confirmed that psychological detachment can act as a “reverse moderator” under challenge stressors—such that its positive effects on innovative behavior disappear when detachment is high [44]. Park et al. similarly noted that for employees who prefer work–life separation, even planned additional work can generate negative outcomes by impeding detachment [45]. Therefore, these findings do not contradict the protective role of psychological detachment; rather, they reveal a “cost” of boundary sensitivity—those who most effectively protect their recovery time may also respond with stronger defensive reactions to external intrusions.

In contrast, psychological detachment showed no significant moderating effect on any pathways involving hindrance‐oriented W_ICTs. This indicates that when communication carries obstructive or exhausting characteristics—such as criticism or unnecessary complications—the resulting resource depletion and behavioral consequences are more direct and pervasive, difficult to buffer through individual detachment alone. Hindrance‐oriented W_ICTs may directly activate stress‐coping mechanisms, making the protective effect of detachment less attainable in such high‐intensity contexts. This aligns with Huo et al., who found that passive communication negatively affects innovation behavior by exacerbating emotional exhaustion [11]. Although passive and hindrance‐oriented communication differ conceptually, both share “involuntariness” and “resource depletion” features. Liu et al. found that when employees perceive low work control, W_ICTs—especially those with hindrance characteristics—significantly increase anxiety and directly lead to cyberloafing [46]. These findings suggest that both communication content and contextual factors jointly shape psychological impact, and that interventions therefore require differentiated strategies tailored to communication type.

## 5. Implications for Nursing Management

The findings hold significant implications for clinical nursing management and nurse self‐management. For managers, it is essential to establish standardized W_ICTs protocols, clearly defining boundaries for nonurgent communication to prevent excessive intrusion into nurses’ rest time [22, 45]. Crucially, organizations should recognize that even developmental communications delivered after hours may prove counterproductive. Simultaneously, institutions should develop training programs focused on psychological detachment. Empirical evidence indicates that interventions such as mindfulness training, leisure skill development, and educational workshops can effectively enhance nurses’ detachment capacity, thereby reducing work stress [47].

For individual nurses, it is important to actively cultivate work–life boundary awareness and consciously practice detachment techniques. This includes engaging in absorbing nonwork activities to facilitate mental unwinding and, crucially, establishing clear boundaries to disconnect from work‐related digital communication after shifts [48]. This practice of strategic disconnection should not be misconstrued as a lack of professionalism; rather, it is a critical investment in cognitive and emotional resources that sustain clinical competence and safeguard patient safety during working hours.

To make such individual efforts feasible and effective, a supportive organizational environment is essential. Healthcare institutions must acknowledge the importance of protecting nurses’ recovery time by establishing clear, collectively agreed‐upon protocols that differentiate between urgent and nonurgent after‐hours communication [49]. This structural support legitimizes personal boundary setting and reduces the ambiguity that often leads to intrusion. Furthermore, cultivating a healthy and respectful communication culture within teams is also critically important [50]. Ultimately, only through these dual safeguards—proactive individual strategies coupled with enabling organizational policies—can the negative effects of W_ICTs be reduced, thereby promoting the long‐term well‐being and stability of the nursing workforce.

## 6. Limitations

This study has several limitations. First, while the multicenter design enhances sample diversity, the use of convenience sampling may limit representativeness. Future studies could use stratified random sampling to improve generalizability. Second, the cross‐sectional design precludes causal conclusions. The observed associations may be influenced by unmeasured third variables, particularly contextual factors such as organizational support or team communication norms. Future longitudinal or experimental studies could help clarify the directionality of these relationships. Third, the classification of challenge‐oriented versus hindrance‐oriented W_ICTs relied on two single items developed as classification indicators rather than as a multi‐item measurement scale. While expert review confirmed the appropriateness of the item content, we acknowledge the inherent psychometric limitations of this exploratory approach—particularly that traditional construct validity assessments are not applicable due to the limited number of items. Accordingly, our subgroup findings should be interpreted as exploratory and hypothesis‐generating. In addition, no correction for multiple comparisons was applied for these exploratory analyses, and these findings warrant confirmation in future studies. Future research should develop a comprehensive multi‐item assessment tool to more objectively measure challenge‐oriented and hindrance‐oriented after‐hours work communication.

## 7. Conclusion

This study indicates that W_ICTs is directly associated with increased work withdrawal behavior among nurses, and this relationship is partially mediated by job burnout. Psychological detachment moderates this relationship by attenuating the effect of W_ICTs on job burnout; however, its protective role is evident only for challenge‐oriented W_ICTs and not for hindrance‐oriented ones. Nursing managers should minimize hindrance‐oriented communication, schedule challenge‐oriented messages strategically, and strengthen psychological detachment systematically, to ultimately reduce both nurses′ job burnout and work withdrawal behavior.

## Author Contributions

X.W. and K.T. made substantial contributions to conception and design, or acquisition of data, or analysis and interpretation of data; Y.G., J.Y., D.L., M.Z., and H.L. involved in acquisition and validation of data; X.W. and K.T. contributed to analysis and interpretation of data; X.W. involved in drafting the manuscript; K.T. participated in revising it critically for important intellectual content.

## Funding

This study was supported by the Scientific Research Fund of Hunan Provincial Education Department (Y20230728), (202401000501), and the Hunan Provincial Natural Science Foundation of China (2026JJ80199).

## Disclosure

All authors gave final approval of the version to be published.

## Ethics Statement

This study was granted approval by the Biomedical Research Ethics Committee of Hunan Normal University (approval no. 2025‐899) on September 6, 2025.

## Conflicts of Interest

The authors declare no conflicts of interest.

## Supporting Information

Additional supporting information can be found online in the Supporting Information section.

## Supporting information


**Supporting Information** STROBE_checklist_cross‐sectional.

## Data Availability

The data that support the findings of this study are available from the corresponding author upon reasonable request.
